# Effect of Transcutaneous Electrical Nerve Stimulation on Gait Parameters in Dogs with Osteoarthritis

**DOI:** 10.3390/ani14111626

**Published:** 2024-05-30

**Authors:** Anja Pedersen, Heli K. Hyytiäinen, Marie Rhodin, Franck Forterre, Johanna Penell, Anna Bergh

**Affiliations:** 1 Department of Clinical Sciences, Swedish University of Agricultural Sciences, 750 07 Uppsala, Sweden; marie.rhodin@slu.se (M.R.); franck.forterre@slu.se (F.F.); johanna.penell@slu.se (J.P.); anna.bergh@slu.se (A.B.); 2 Department of Equine and Small Animal Medicine, Faculty of Veterinary Medicine, University of Helsinki, P.O. Box 57, 00014 Helsinki, Finland; heli.hyytiainen@helsinki.fi

**Keywords:** TENS, pressure sensitive mat, locomotion, lameness, electrotherapy, kinetic, canine, pain, rehabilitation, musculoskeletal system

## Abstract

**Simple Summary:**

Although scientific evidence for treatment efficacy is lacking, transcutaneous electrical nerve stimulation is used in dogs as a pain-relieving treatment. This randomised single-blinded cross-over study aims to investigate whether treatment with transcutaneous electrical nerve stimulation will affect gait parameters in dogs with osteoarthritis. Fifteen dogs were included in the study, and all dogs were over one year of age, lame, and had chronic pain for more than three months. The dogs were treated with transcutaneous electrical nerve stimulation for seven or ten days, and their gait pattern in trot was evaluated with a pressure-sensitive mat. In the present study, no significant differences were seen between transcutaneous electrical nerve stimulation and placebo treatments for any of the gait parameters evaluated by the pressure-sensitive mat. Further studies are needed to confirm the observations.

**Abstract:**

Osteoarthritis is a common degenerative disease in dogs, often manifested as pain, joint swelling, and lameness. Despite the lack of scientific evidence for its treatment efficacy, transcutaneous electrical nerve stimulation (TENS) is used in dogs as a pain-relieving treatment. This randomised single-blinded cross-over study investigated the effect of TENS on gait parameters in fifteen dogs with osteoarthritis. Stance time, swing time, stride time, stride length, peak vertical force (%BW), vertical impulse (%BW*sec), and symmetry indices were obtained using a pressure-sensitive mat. TENS treatment of 80 Hz and 100 µs with an individually selected amplitude was conducted for 45 min once daily for a treatment period of seven or ten days. No significant differences were seen between TENS and placebo for any of the gait parameters. Hence, in this study, TENS did not affect gait parameters, compared to placebo. Further studies are needed to confirm the observations.

## 1. Introduction

Osteoarthritis (OA) is a common degenerative disease in dogs, with a possibly long-term need for therapy [1,2,3,4]. It is usually manifested as pain, joint swelling, and reduced joint mobility, causing varying degrees of lameness [5,6,7]. Joint pain may lead to pain-induced functional impairment, regarded as one of the clinical signs of OA [8]. There are several treatment strategies for OA, including pharmaceuticals, nutraceuticals, weight reduction, regenerative medicine, therapeutic exercises, and different rehabilitation modalities [9,10,11,12,13,14]. It is likely that the management of canine OA may benefit from an integration of both pharmacologic and non-pharmacologic treatments. Common pharmaceuticals for OA are nonsteroidal anti-inflammatory drugs (NSAIDs), corticosteroids, and monoclonal antibodies [11,15,16,17]. However, in 55% of the studies on NSAIDs, adverse effects are reported [18]. Even if the majority of adverse effects are mild, they may restrict long-term use of medication [15,17,19,20,21,22]. Further, concurrent disease may also restrict the use of corticoids [15,16,20]. Untreated pain causes suffering for the dog and has a negative impact on its welfare, as well as on the wellbeing of the owner, since managing a dog with chronic pain negatively affects their life [23]. Therefore, it is relevant to study non-pharmacologic treatments, such as different rehabilitation techniques, as complementary treatments, but especially as stand-alone treatments for those dogs that do not tolerate NSAIDs or corticosteroids and where treatment with monoclonal antibodies is not feasible.

Veterinary rehabilitation has attracted increased interest from dog owners and animal health staff in recent decades. Rehabilitation is considered an important component of an overall long-term treatment strategy for OA. Among different rehabilitation modalities, there is an increasing use of transcutaneous electrical nerve stimulation (TENS). TENS is a device that uses electric current, delivered though electrodes on the skin, to stimulate nerve fibers for therapeutic reasons, i.e., as pain relief. The specific treatment settings include adjustable parameters such as pulse frequency, pulse duration, and intensity. TENS is claimed to provide pain relief through either endogenous opioid release (low-frequency TENS) or on a segmental level by the use of the pain gate theory (high-frequency TENS) [24,25]. The latter is believed to be effective by applying stimuli to large diameter non-noxious afferents (A-beta), which subsequently reduces pain via decreased nociceptor activity [24,25]. Further, studies have shown an increase in β-endorphins and methionine-enkephalin in human subjects, a release of glutamate and substance P in animals with inflammation, neuropathic, or incisional pain, a reduction in pressure pain thresholds at the site of TENS and at sites outside the area of application, and a reduction in microglia and astrocyte activation in the spinal cord in both osteoarthritic and neuropathic pain animal models [26,27,28,29].

In humans, TENS is used as a pain-relieving treatment and a complementary or single treatment for OA [24,30,31,32]. A systemic review and meta-analysis of TENS for acute and chronic pain in humans, based on 381 studies, concluded that there was moderate-certainty evidence that pain intensity was lower during or immediately after TENS treatment compared to placebo [33]. The review included studies that used participant-reported strong but comfortable TENS sensation stimulation, with electrodes at the site of pain or over nerve bundles proximal to the site of pain. The effect was evaluated directly after treatment and with different types of pain scales [33]. However, other studies report no effect on pain compared to control [34,35,36,37]. Conflicting results and, thus, inconclusive evidence are explained by the low quality of relevant studies as well as the diversity in treatment protocols [37]. Regarding animal studies, studies report that TENS produced an analgesic effect in rodents with experimentally induced OA [38,39]. The scientific documentation on the effect of TENS in dogs is even sparser than in humans and laboratory animals. Thus, several authors report that there is a need for more canine studies [40,41,42,43]. The results from the few existing studies indicate that treatment with TENS may increase weight bearing on the affected limb in dogs with OA for up to 180 min, with the greatest significant difference immediately after treatment [42]. A study on dogs with canine ankylosing spondylitis showed a decrease in signs of pain evaluated by visual analogue scale and clinical examination after TENS treatment [40]. Further, a weight-reduction study on dogs with OA examined the difference in lameness in two treatment groups, both with dietary protocol, but with two different physical therapy programmes, one of which included TENS treatment. Results indicate that dogs that received an additional TENS treatment showed significant improvement, evaluated with force plate and changes in peak vertical force (PVF) and vertical impulse (VI), whereas dogs with no TENS treatment showed only significant improvement after 4 months [41,44]. Two of the previous studies have evaluated the effect of TENS by the use of kinetic techniques, i.e., pressure-sensitive mats and force plates; the latter is regarded as the gold standard for measuring ground reaction forces [45,46,47,48,49,50,51,52,53]. Recent studies have compared the results from these two kinetic techniques and report that they are equally reliable but not interchangeable [54,55,56,57]. Further, studies have shown a high agreement between repetitive measurements in individual dogs [58]. The use of these techniques enables the registration of different gait parameters, such as temporospatial parameters, peak vertical force (PVF), vertical impulse (VI), and symmetry indices (SIs) [7,56,59,60,61,62]. PVF and VI adjusted to bodyweight (% BW) show a low variability [56,60,61]. Thus, the kinetic techniques contribute, together with an orthopaedic examination, to a more objective lameness evaluation. 

In OA, mild to moderate lameness is often seen, and kinetic studies show alterations in PVF and VI, as well as symmetry indices [7,47,63,64,65,66]. Studies on pain-relieving treatment of dogs with OA have used changes in PVF and/or VI as outcome measures, showing therapeutic effects such as an increase in load on the lame limb but also redistribution of weight to other limbs [45,67,68,69,70,71,72,73,74]. Further, registration of temporospatial parameters has been used, but results are rarely described [59,60,75]. 

Due to the increasing clinical use of TENS, together with the lack of research on its possible effects, the present cross-over study investigates the effect of TENS on canine gait parameters, evaluated with a pressure-sensitive mat. The null hypothesis is that, for dogs with OA, treatment with TENS will not affect gait parameters differently than placebo treatment.

## 2. Materials and Methods

**Figure 1:**
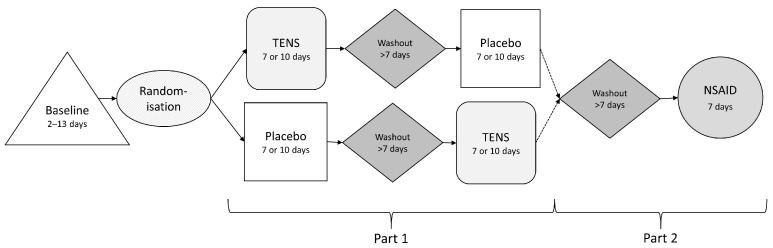
Schematic view of the study. The difference in length for the TENS and placebo treatment (7 or 10 days) depends on whether the dog participated in the pilot study (7 days).

The study consisted of two parts—part 1 (TENS and placebo intervention) and part 2 (an NSAID intervention)—see Figure 1. For the comparison of TENS and placebo treatment effect, a prospective, single-blinded, randomised, placebo-controlled, and cross-over design was utilised (Figure 1). A pilot study was conducted in order to test the study design (study part 1), consisting of seven days of treatment with TENS or placebo performed by animal health personnel, and the pilot data were included in the final data. For the evaluation of the effect of NSAIDs (study part 2), a one-group pre-test–post-test study design was used (Figure 1).

Client-owned dogs of any sex or breed with confirmed OA were eligible for the study. Recruitment of dogs was conducted through social media (Facebook), through email posting and advertising in magazines, and at veterinary practices in the local area. Dogs were included if they were over 1 year of age, were 1–3 degrees lame in trot on a 5-degree scale at an orthopaedic examination, had an OA diagnosis confirmed by diagnostic imaging and had had chronic musculoskeletal pain (>3 months), diagnosed by a veterinarian before the study [76]. If the dog was diagnosed with OA in multiple joints, enrolment was based on the worst affected limb (referred to as the “lame limb”) based on the dog’s clinical history together with a clinical assessment and baseline/preintervention performed kinetic measurements.

Dogs were excluded if they had a metallic implant that interfered with treatment, a pacemaker or a tumour in the treatment area, or a sensory deficiency in the treatment area. The latter was assessed by palpation of the whole body and by manually stroking the skin at the selected localisations of the electrodes. Dogs were excluded from part 2 (NSAID intervention) of the study if they had a history of adverse reactions to NSAIDs.

**Figure 2:**
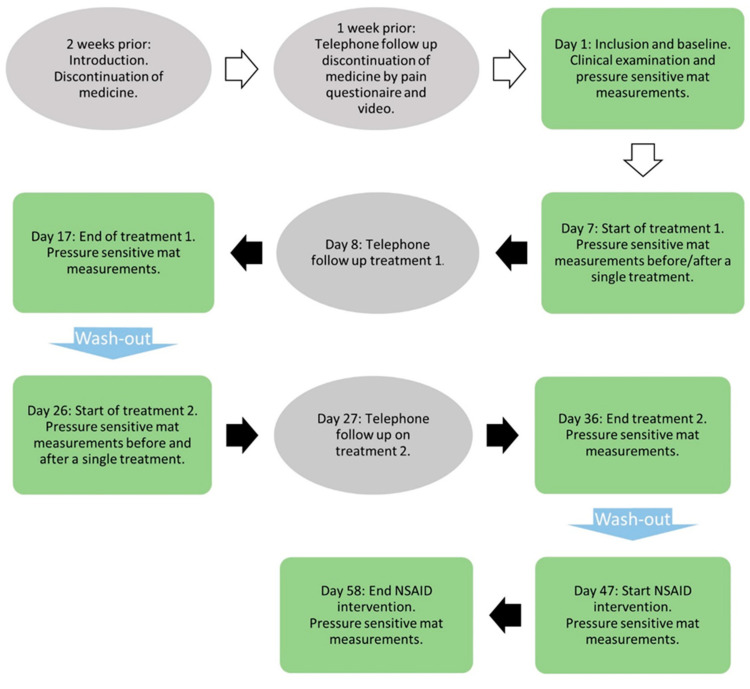
Study design and protocol. Grey ellipse = telephone contact. Green rectangles = physical visit. Black arrows = treatment period. Blue arrows = washout period.

Informed consent from the owners was signed and an ethical permit was granted by a source (information withdrawn as a result of blinding), and the study was performed according to guidelines established in the Helsinki Declaration [77]. The study included five to seven visits (measurement occasions) to the research facility, depending on participation in the NSAID part of the study (Figure 2). Registration on the pressure-sensitive mat was performed at each visit to the research facility. Data collection took place between September 2018 and January 2020.

### 2.1. Evaluation Methods

The dogs were evaluated via clinical examination, pressure-sensitive mat measurement and pain assessment questionnaires (Helsinki Chronic Pain Index and Canine Brief Pain Index). The pain index assessments were used during the baseline and throughout the study period to ensure animal welfare, especially after an eventual discontinuation of medication. In this article, results from the pressure-sensitive mat are presented. 

#### 2.1.1. Pressure-Sensitive Mat

A pressure-sensitive mat “Walkway High Resolution HRV4” (Tekscan Inc., Norwood, MA, USA) and software “Walkway Research ver. 7.60-31” (Tekscan Inc., Norwood, MA, USA) were used to collect the kinetic data. The measurements were made within an hour after the first treatment session (from now called “single treatment”) and within 12–24 h after the last treatment of the whole treatment period (“multiple treatments”). The mat was regularly calibrated, and the calibration files used were coherent with each dog’s weight. 

The mat (195 × 45 × 0.57 cm) was placed in a corridor next to a wall and was covered with a 1 mm-thick non-slip plastic mat. Cameras filmed the dogs from a lateral and a craniocaudal aspect. The dogs trotted over the pressure-sensitive mat at a comfortable individual pace. The same handler and handler side was used in the absolute majority of measurements. A valid trial was defined as the dog’s correct behaviour over the mat and the number of step cycles (a minimum of two step cycles/eight stances). Correct behaviour was defined as the dog trotting at a constant pace in a straight line, looking straight ahead with minimal intervention from the handler. It was subjectively assessed by the author(s) and noted in the data collection protocol. The criteria for successful kinetic data collection were three trials in trot (a 2-beat gait with left front (LF)/right hind (RH)-suspension-right front (RF)/left hind (LH) steps), with a velocity of between 1.5 and 2.2 m/s and an individual variance of <0.5 m/s. 

The following gait parameters were registered: stance time, swing time, stride time, stride length, peak vertical force (%BW), vertical impulse (%BW), and symmetry indices based on peak vertical force. 

#### 2.1.2. Clinical Examination

To investigate if the dog met the inclusion criteria, a clinical examination was conducted by an experienced veterinarian. 

#### 2.1.3. Pain Questionnaires

Helsinki Chronic Pain Index and Canine Brief Pain Index were used as a control for animal welfare, especially after the discontinuation of pain-relieving medication [78,79,80,81]. The two pain questionnaires were answered by the owner or another person who had daily contact with the dog. The respondent was instructed to fill out the forms once a week to keep track of the dog’s pain score and to contact the authors if the dog showed signs of deterioration. 

### 2.2. Study Protocol

Telephone contact with eligible dog owners was made at both two weeks and one week before the start of the study. The suitability of the dogs was determined by the information the owners sent in when they expressed interest in the study. The first call focused on the retrieval of the dog’s status and medication (Figure 2). Owners were asked to send in videos of their dog’s locomotion from a lateral and cranial view in trot for an initial lameness assessment. Based on the video and the phone information, the study veterinary surgeon assessed if the dog’s pain medication could be discontinued. Pain medication was reinstated before baseline if deemed necessary by the same veterinarian, based on pain questionnaires and owner information. For these dogs, pain medication was given throughout the study. If the medication was needed later in the study (i.e., after baseline), the dogs were excluded. The dog’s status was checked one week prior to the start of the study via the second telephone call, pain questionnaires, and new videos. Owners could make additional contact with the study team when needed. 

In part 1 of the study, each dog was allocated randomly into either TENS or placebo treatment for the first treatment period. The treatments were reversed during the second treatment period (Figure 1). A washout period of a minimum of ten days was used between the two treatment periods in study part 1. Part 2 of the study started after a washout period for those dogs that could withstand NSAID treatment. The NSAID intervention consisted of a seven-day treatment.

#### 2.2.1. Transcutaneous Electrical Nerve Stimulation and Placebo Treatment

Treatment was administered with either one of two TENS machines—Profile TENS (Body Clock Health Care Limited, London, UK) or a Cefar TENS Chattanooga (Enovis, Lewisville, TX, USA)—using CEFAR coal fibre electrodes (3 × 5 cm) (Enovis, Lewisville, TX, USA). The treatment programmes of the two TENS devices were synchronised so that the settings were identical. The skin was clipped precisely where the electrodes were situated during treatment, soaked with water and ultrasound gel was used as a transmitting substance. Two electrodes, a minimum of 4 cm apart, were placed on intact skin, with electrodes at the site of pain (distal placement) or over nerve bundles proximal to the site of pain (proximal placement). 

The first treatment was conducted partly by animal health staff and partly by the owner under the supervision of the animal health staff. During the following treatments, the dogs were treated by their owners, except for the pilot study, where the treatments were conducted by animal health personnel. The treating person, mainly the owner, received instructions both verbally and in writing before each treatment period (TENS and placebo) regarding how to perform the treatment. Optional additional supervision was offered by one of the authors. 

The TENS device was set to a constant current with a frequency of 80 Hz and a pulse duration of 100 µs based on previous studies and clinical experience. The intensity (amplitude, unit milliampere (mA)) was increased until muscular fasciculation in the treatment area’s muscles occurred and was lowered if the dogs expressed discomfort. The intensity was then gradually increased during the treatment session to maintain sensation throughout. The treatment sessions were 45 min once daily. The treating person kept a diary of each treatment session, including treatment duration, electrode placement, used intensity, and behaviour of the dog. The placebo treatment protocol was identical to the TENS treatment, with the exception that the device was not switched on. Each treatment period’s length was ten days, except for the pilot study, where the dogs were treated for seven days. 

#### 2.2.2. NSAID Treatment

Firocoxib was administered orally by the owner once daily for seven days after the finalisation of the TENS part of the study. A dosage of 5 mg per kg body weight was subscribed based on the recommended dosage by the manufacturer [82]. Owners were instructed to start the medication eight days before the final measurements of the NSAID treatment. 

### 2.3. Data Management and Statistical Analysis

For all gait parameters, an average value based on three trials over the mat (a minimum of six step cycles/twenty-four stances) was included. If there were not three trials that met the inclusion criteria, the average value of two trials was used. 

Three different symmetry indices (SIs) were used; either body quadrants, body sides, or body halves were compared. For the commonly used SI_limb_, the quadrant containing the lame limb was compared to the contralateral quadrant, i.e., the sound limb, and the SI_sagittal_ was compared to the left and the right sides of the body. The additional SI_transversefront_ compared front limbs from front limb lame dogs with their sound hindlimbs, and the SI_transversehind_ compared hindlimbs from hindlimb lame dogs with their sound front limbs.

SIs were calculated from peak vertical force (%BW) by using the following equation:

$$\begin{array}{l} {\mathrm{SI}}_{\mathrm{limb}}=\frac{\mathrm{lame}\;\mathrm{limb}}{\mathrm{contralteral}\;\mathrm{sound}\;\mathrm{limb}}\\ {\mathrm{SI}}_{\mathrm{sagittal}}=\frac{\mathrm{front}\;\mathrm{and}\;\mathrm{hindlimb}\;\mathrm{from}\;\mathrm{lame}\;\mathrm{body}\;\mathrm{side}}{\mathrm{front}\;\mathrm{and}\;\mathrm{hindlimb}\;\mathrm{from}\;\mathrm{sound}\;\mathrm{body}\;\mathrm{side}}\\ {\mathrm{SI}}_{\mathrm{transversefront}}=\frac{\mathrm{front}\;\mathrm{limbs}\;\mathrm{from}\;\mathrm{lame}\;\mathrm{body}\;\mathrm{half}}{\mathrm{hindlimbs}\;\mathrm{from}\;\mathrm{sound}\;\mathrm{body}\;\mathrm{half}}\\ {\mathrm{SI}}_{\mathrm{transversehind}}=\frac{\mathrm{hindlimbs}\;\mathrm{from}\;\mathrm{lame}\;\mathrm{body}\;\mathrm{half}}{\mathrm{front}\;\mathrm{limbs}\;\mathrm{from}\;\mathrm{sound}\;\mathrm{body}\;\mathrm{half}} \end{array}$$

Differences in gait parameter values before and after treatment with TENS and placebo, respectively, were compared. The comparison was made on data collected before and after the first treatment session and before and after the last day of the whole treatment period for TENS and placebo; hereafter, the terms “single treatment” and “multiple treatments” are used. Further, differences in gait parameters were compared before and after the last day of NSAID treatment. 

The data were compiled in Excel (Microsoft Excel 2016 (16.0.5443.1000), Microsoft Corporation, Redmond, WA, USA), and the statistical analysis was made in R (version 4.1.2 (2021-11-01)—”Bird Hippie”, R Core Team, Vienna, Austria). The individual data and history of disease of the participating dogs are presented descriptively. The pressure-sensitive mat data were analysed in a linear mixed-effects model for a 2 × 2 cross-over design for the TENS and placebo part, with gait parameters (stance time, swing time, stride time, stride length, peak vertical force (%BW), vertical impulse (%BW*sec), and symmetry indices) as continuous outcomes. For the NSAID intervention, a linear regression model was used with gait parameters (stance time, swing time, stride time, stride length, peak vertical force (%BW), vertical impulse (%BW*sec), and symmetry indices) as continuous outcomes. In the linear mixed effects model, dog was set as a random effect, and age, sex, weight, simultaneous NSAID treatment, and velocity were set as fixed effects for all parameters except for the symmetry indices where age, velocity, and simultaneous NSAID treatment were excluded from the fixed effects due to restriction of numbers of factors in the model. Residuals were normally distributed. The significance level was set to *p* < 0.05. 

## 3. Results

### 3.1. Descriptive Statistics

A total of 38 dogs were initially selected for the study. Of these, 26 matched the inclusion criteria and were enrolled in the study, and data from 15 of these dogs were finally used in the study. Of the 26 enrolled dogs, two were lost to follow-up, five were excluded due to unconfirmed diagnostic imaging diagnosis of OA, and two had to be excluded due to data corruption. Furthermore, two dogs had to be excluded: one due to an aggravated caudal cruciate ligament injury noted during baseline and the other due to the illness of the owner after one treatment period. One dog did not participate in part 2 of the study (NSAID intervention) due to a traumatic fracture of the elbow, so data from part 1 of the study were used. During part 2 (NSAID intervention), one dog needed to end the medication after five days due to suspected adverse reactions to the treatment, so the data from the NSAID intervention were excluded from the study.

The descriptive data of the dogs are presented in Table 1. The mean age was 6.8 years (±1.9 years). The mean weight was 22.7 kg (±9.4 kg). There were five mixed breeds: three Labrador retrievers and one each of Australian Cattle Dog, Beagle, Border Collie, Flatcoated Retriever, Malinois, medium-sized Poodle, and Staffordshire Bull Terrier. In part 1 (TENS and placebo intervention), one of the dogs received 7 days of treatment, and 14 dogs received 10 days of treatment. Two of the dogs were treated with NSAIDs during the whole study. Electrodes were placed at the site of pain (distal placement) in 14/15 dogs; in one dog, a placement over nerve bundles proximal to the site of pain (proximal placement) was made. 

### 3.2. Gait Parameters

The data were collected from a total of 108 measurement occasions (visits to the facility). Three trials per dog were included from 105 of the 108 measurement occasions. For the remaining three occasions, two trials (a minimum of four step cycles/sixteen stances) were used due to incomplete registrations. The mean value for a trial for all the dogs was 8.8 (range 8–12) stances, which corresponds to two step cycles. The same handler was used for 103 of 108 measurement occasions, and the handler was on the same side of the dog in 104 out of 108 occasions. The dogs trotted over the pressure-sensitive mat between 2 and 20 times (trials) on each measurement occasion. 

No significant differences were seen between TENS and placebo treatments for stance time, swing time, stride time, stride length, peak vertical force (% BW) and vertical impulse for (% BW*sec) for any of the limbs. Similarly, no significant differences were seen, comparing before and after NSAID treatment, for stance time, swing time, stride time, stride length, and peak vertical force (% BW). However, the results show a significant increase in vertical impulse (% BW*sec) for the ipsilateral limb (*p* = 0.02). Estimated mean values and *p*-values for the gait parameters can be seen in Table 2.

No significant differences were seen for the SIs, either for single or multiple treatments, between TENS and placebo treatments. The NSAID treatment period showed no significant difference between before and after for any of the SIs. The mean values and significance for the symmetry indices can be seen in Table 3. 

**Figure 3:**
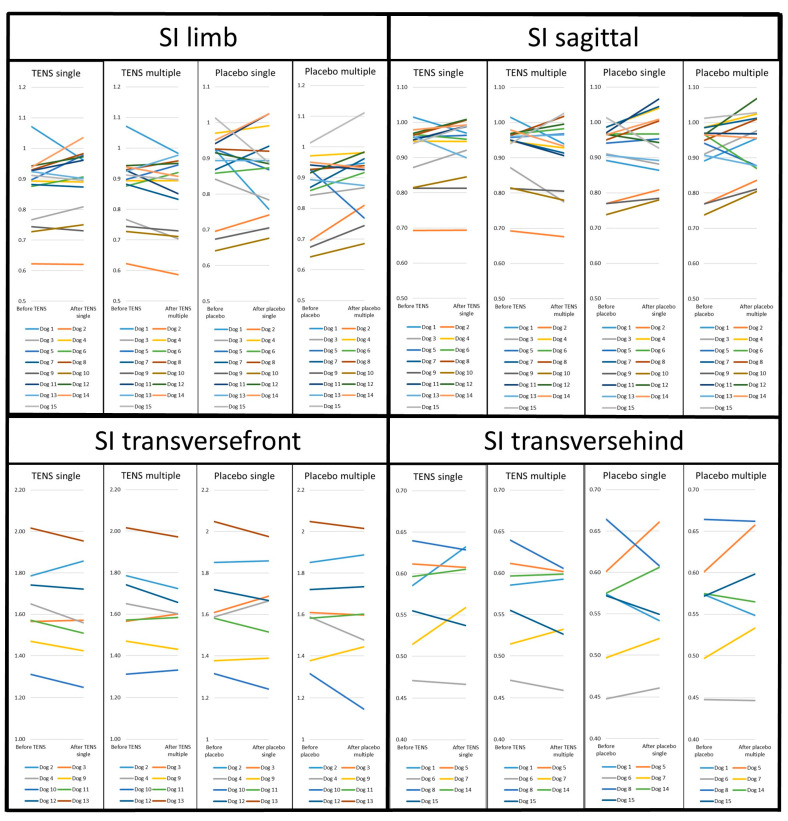
Individual symmetry indices of peak vertical force (%BW) for TENS and placebo. SI = symmetry index. SI_limb_ = lame limb/contralateral sound limb. SI_sagittal_ = front and hind limb from lame body side/front and hind limb from sound body side. SI_transversefront_ = front limbs from lame body half/hindlimbs from sound body half. SI_transversehind_ = hindlimbs from lame body half/front limbs from sound body half. Single = single treatment. Multiple = multiple treatments. The suggested reference values are the following: SI_limb_ = 1.0, SI_sagittal_ = 1.0, SI_transversefront_ = 1.5, and SI_transversehind_ = 0.66.

In order to further visualise the individual differences between TENS and placebo, the individual SI values are presented in spaghetti plots in Figure 3.

## 4. Discussion

Our results show no significant differences in peak vertical force (%BW), vertical impulse (%BW*sec), or SIs of osteoarthritic dogs when treated with TENS compared to placebo. Nor were there any significant differences in temporospatial parameters, such as stance time, swing time, stride time and stride length. Accordingly, our null hypothesis was accepted for this study protocol and population. 

Our result differs from the only previous study on TENS as a stand-alone treatment for OA in dogs, where five dogs significantly increased weight bearing on the affected limb, evaluated by a force plate, indicating a positive pain-relieving effect of TENS [42]. The dogs were treated with a single treatment, at 70 Hz for 20 min, and the largest increase in weight bearing on the affected limb was seen immediately post-treatment and with changes remaining up to 180 min [42]. Similar to the Johnston et al. (2002) study, our measurements after a single treatment were made within an hour post-treatment [42]. Thus, the results from the present study are not in accordance with the Johnston et al. (2002) study nor with human studies indicating a pain-relieving effect during and shortly after TENS treatment [30,42]. 

The present study called for dog owners with a large commitment since it required a high degree of involvement from the owners, which narrowed the availability of possible candidates. Still, the study population in the present study was larger than in the previous study. It consisted of dogs with various locations of arthritic joints, thus representing the diverse patient population with OA. However, the five dogs in the Johnston study (2002) had OA in the stifle and the fifteen from our study in various joints, which may have had an effect on the lameness pattern and thus the inconsistent results [42,53,83].

Even though there are similarities in study design between previously conducted studies and the present study, there are also differences. A cross-over design was used to study the effect of the TENS and placebo interventions. This design entails a higher power and more statistical efficiency than the parallel design without control groups that have been used in previous studies on TENS in dogs [40,42,84]. An additional difference between our study and the previous ones was that two dogs were treated with NSAIDs throughout the TENS and placebo intervention. This was accounted for in the statistical analysis, with concurring NSAID treatment set as a fixed effect, and should not have affected the results significantly. 

Our treatment sessions were longer than the treatment in the studies by Johnston et al. (2002) (20 min), Mlacnik et al. (2006) (15 min) and Krstić et al. (2010) (15 min) [40,41,42]. The decision to have a longer treatment session was based on clinical experience and from studies on humans, indicating, for example, an optimal treatment length of 40 min in knee OA [32,33,37,85]. In our study, a frequency of 80 Hz was used, a setting in between the frequency used in the studies by Krstić et al. (2010) (85 Hz) and Johnston et al. (2002) (70 Hz) [40,42]. A low degree of consistency in treatment settings was highlighted as one major limitation of TENS-related studies in a systematic review by Gibson et al. (2019) and Hyytiäinen et al. (2023) [37,43]. Therefore, our study aimed to have similar settings for frequency as the previous studies in dogs, and 80 Hz was used [37,40,42]. Further, the use of the strongest comfortable intensity possible is critical for pain relief with TENS; therefore, the intensity in the present study was increased until muscular fasciculation occurred as long as the dogs would withstand it [24,37]. Also, increasing intensity during the treatment compared to keeping the intensity fixed has been shown to decrease analgesic tolerance after five days in rats [38]. Since our study lasted longer than five days, the intensity was increased during treatment. As recommended by the literature, the most common electrode placement in our study was at the painful site; however, in one dog, this was not possible due to a limited area for electrode placement [25,86,87]. A transferred analgesic effect has been shown to happen in humans, and therefore, the placement over proximal nerve bundles is regarded as a suitable electrode location [88]. 

High-frequency TENS is claimed to alleviate pain through the pain gate theory and endogenic opioid release [24,38,39,89,90]. Studies on other pain-relieving treatments of dogs with OA have used changes in PVF and/or VI as outcome measures, evaluated with kinetic techniques [44,45,67,68,69,70,71,72,75]. Thus, therapeutic effects have been evaluated as an increase in weight on the affected limb and as a redistribution of weight to other limbs [59,60,74,75,91]. Measurement of ground reaction forces with a pressure-sensitive mat technique is an objective method for detecting asymmetries in weight distribution and takes all four limbs into consideration by using SIs [50,58,63,83,92,93,94,95,96]. In a previous study, when measuring 115 lame dogs on a pressure-sensitive mat, a specificity of 84.6% and a sensitivity of 91.1% were determined [50]. However, whether the technique can be used in the diagnosis of OA in dogs is discussed since studies have shown an overlap in the values for ground reaction forces of sound dogs and dogs with OA [58]. Further, there are several suggested cut-off values for the distinction between lame and not lame, indicating the difficulties in using the technique for the determination of a diagnosis [58,63,97,98]. The kinetic registrations in the present study were used to detect eventual changes in gait parameters within an individual dog and not for diagnosing OA; therefore, no cut-off values have been used. 

Peak vertical force is considered an accurate variable for detecting weight distribution between limbs and is often used together with vertical impulse in gait analysis [56,58,63]. Peak vertical force and vertical impulse have been shown to be consistent in dogs with OA over time. Over two months, a change of 5% in these values is unusual, and a change of 10% is rare. Therefore, an effective treatment for OA could be expected to provide more than a 5% change in PVF and VI, which was not the case for TENS treatment compared to placebo in our study [56,68,99]. Besides ground reaction forces, temporospatial parameters such as stance time, swing time, stride time and stride length may be used to evaluate lameness; however, the documentation is limited [58,92,100]. The temporospatial parameters were included in the present study as the data can form a basis for future research in the area.

In the present study, some of the dogs had OA in multiple joints of multiple limbs. In these cases, the most affected limb (i.e., lame limb) was determined based on clinical history, clinical examination and kinetic data as in the studies by Moreu et al. (2003), Madore et al. (2007) and Roush et al. (2010) [83,101,102]. However, the involvement of multiple locations of OA could have influenced the results based on the reasoning that the dog would not shift as much of its weight from the affected limb to other limbs as if there was a single joint involvement [103,104,105]. However, in our study, gait parameters for all limbs and SIs were analysed, which most likely would have detected a difference in the exerted pressure on the ground from any limb based on the sensitivity of the pressure-sensitive mat [41,58,71,104]. The SIs in the present study are indices comparing the PVF between limbs and, thus, less vulnerable to the influence of velocity than solely reported PVF data, with SI sagittal showing a low variability of 2–3% in sound dogs [56,95,106]. The use of SIs to complement other parameters is becoming more common [41,56,61]. There is an increasing body of evidence from equine studies and some from canine regarding the influence on weight distribution and motion symmetry from compensatory lameness [74,91,100,103,107,108,109]. Thus, the reasons behind the inclusion of additional SIs, SI transverse and SI ipsilateral, were twofold: first, to get a better picture of eventual changes in the weight distribution between all four limbs, and second, to supply information for further research in the area. Based on the SIs, the results from the present study are not indicative of a TENS treatment effect different from that of a placebo.

As previously stated, velocity has a major effect on all gait parameters except for the SIs [110]. Therefore, the velocity was set as a fixed effect in the analysis for the TENS and placebo’s effects on the gait parameters, with the exception of SIs. Furthermore, a fixed velocity interval was specifically used for the selection of trials [56,111]. Three trials were included for each measurement occasion, which is considered the gold standard, and this was possible most of the time [56]. In the three remaining occasions, the registrations were cancelled after two valid trials due to the risk of deterioration in lameness [56,112]. It is unlikely that the lack of three missing trials out of over three hundred had any major influence on the results. Mickelson et al. (2017) showed a mild alteration in weight bearing with repeated measurements in 61 dogs with mild to moderate lameness; the variance was <5% [112]. Therefore, in our study, the three missing trials should not have influenced our results drastically.

The handler may affect the outcome of the pressure-sensitive mat measurements by his placement in relation to the dog and through his general behaviour [113,114]. In the present study, the majority of trials had the same handler and leash side. According to Jevens et al. (1993), a variation between 0 and 7% of the total variance in gait parameters is to be expected when changing handlers [113]. However, due to the small portion of measurements that was affected by the change of handler and side, this should not have influenced the results significantly.

All the dogs included in the present study had OA and pain from the musculoskeletal system as determined by clinical history and clinical examinations. Medication with NSAID is used as the gold standard when investigating the effects of new pain-relieving treatments [115]. The NSAID intervention, i.e., study part 2, was performed to test whether a standard pain relief medication would change the gait parameters of the dogs. No significant differences were seen before and after NSAID treatment for stance time, swing time, stride time, stride length, and peak vertical force (%BW). However, the results show a significant increase in vertical impulse (%BW*sec) for the ipsilateral leg (*p* = 0.02) after treatment with NSAID. Previous studies on weight redistribution have not shown an isolated increase in VI for the ipsilateral leg without any other changes in gait parameters for the other limbs [74,100,107]. Our result is, therefore, not consistent with the current documentation on weigh redistribution in lame dogs and can, therefore, be suspected to be a false positive value. Firocoxib has an indication for pain relief in OA, with the dosage per kg body weight given in the study; however, the length of the treatment for sufficient pain relief is not specified [82]. The treatment period might have been too short to ensure increased efficacy in dogs with chronic pain [21,116,117]. It is also possible that the discrete change in VI (%BW) is explained by the time (12–24 h) between the last medication and the measurement occasion [82]. Further, in a study by Rhodin et al. (2017), horses with lameness did not change their gait pattern in response to NSAID treatment; however, after diagnostic anaesthesia, the lameness improved, so the minor response in the present study may also be due to the insufficient treatment effect of the NSAID [118].

In both veterinary and human medicine, the evidence of the effect of TENS on chronic pain is inconclusive, reported in systematic reviews [35,37,43,119,120,121]. The major deficits in the scientific material are small studies of low quality and a large variety of settings used for the TENS treatment. Future studies in veterinary medicine should, therefore, ensure similar treatment protocols and study designs to be used to increase the level of evidence.

## 5. Conclusions

To the author’s knowledge, our study is one of the few that measures the effect of TENS treatment in dogs with OA. The results of our study provide preliminary evidence that TENS, with the settings used, did not cause significant changes in gait parameters in dogs with OA. Thus, the null hypothesis that TENS treatment of dogs with OA will not change gait parameters differently than placebo treatment was accepted. However, further studies are needed to confirm the clinical efficiency of TENS as a treatment for OA in dogs. 

## Figures and Tables

**Table 1:** Descriptive data of the dogs. OA = osteoarthritis, LF = left front limb, LH = left hindlimb, RF = right front limb, RH = right hindlimb, BF = both front limbs, BH = both hindlimbs, DP = distal placement electrodes, PP = proximal placement electrodes, L7S1 = 7th lumbar vertebra and sacrum.

Dog	Age (Years)	Breed	Weight (kg)	Lameness at Inclusion	Diagnosis and Electrode Placement	Number of Days of Treatment	NSAID Treatment through the Whole Study
Dog 1	8	Beagle	13	1° LH	OA stifle LH. Cruciate ligament injury LH. DP.	7	No
Dog 2	8	Labrador Retriever	31	2° LF	OA metacarpal joint phalanx 4 and 5 LF, phalanx 5 RF and elbow LF. DP.	10	Yes
Dog 3	6.5	Poodle, medium size	7	1° LF	OA elbow LF. DP.	10	No
Dog 4	8	Malinois	27	1° LF	Moderate OA shoulder LF. Mild OA shoulder RF. Disc herniation L7S1. DP.	10	No
Dog 5	3	Mixed breed	15	1° LH	OA stifle LH. DP.	10	No
Dog 6	8	Mixed breed	41	2° RH	OA stifle BH. DP.	10	No
Dog 7	6	Mixed breed	18	1° LH	OA hip BH. DP.	10	No
Dog 8	8	Border Collie	16	1° RH	OA lumbar spine. OA shoulder and phalanx BF. PP.	10	No
Dog 9	8	Labrador Retriever	37	3° RF	OA carpus and phalanx BF. OA hips BH. DP.	10	Yes
Dog 10	5	Mixed breed	17	3° LF	OA elbows and phalanx BF. Spondylosis spinal cord. DP.	10	No
Dog 11	7	Flatcoated Retriever	26	1° LF	OA carpus RF. Lameness LF. DP.	10	No
Dog 12	2	Labrador Retriever	30	1° LF	Fragmentation of processus coronoideus medialis elbow LF. OA elbow LF. DP.	10	No
Dog 13	7	Staffordshire Bull Terrier	13	1° LF	OA stifle LH. Operated cruciate ligament injury BH. Elbow dysplasia grade 2 BF. Hip dysplasia BH. DP.	10	No
Dog 14	9	Mixed Breed	30	1° LH	OA hips BH. OA lumbar spine. DP.	10	No
Dog 15	8	Australian Cattle Dog	20	1° LH	OA tarsus LH. DP.	10	No

**Table 2:** Estimated mean values, number of dogs and *p*-value for the different gait parameters. SD = standard deviation. N = number of dogs. Sec = seconds. Cm = centimetre. %BW = percentage of body weight. Single = measurement after a single treatment. Multiple = measurement after multiple treatments. ** = significant *p*-value (*p* < 0.05).

Parameter	Time	Leg	TENS (Mean ± SD)	N	Placebo (Mean ± SD)	N	*p*-Value	NSAID (Mean ± SD)	N	*p*-Value
Stance time (sec)	Before	Lame	0.19 ± 0.04	15	0.20 ± 0.04	15		0.18 ± 0.04	9	
		Contralateral	0.20 ± 0.04	15	0.20 ± 0.04	15		0.19 ± 0.03	9	
		Ipsilateral	0.19 ± 0.04	15	0.20 ± 0.04	15		0.19 ± 0.05	9	
		Diagonal	0.20 ± 0.04	15	0.20 ± 0.04	15		0.19 ± 0.05	9	
	After single	Lame	0.20 ± 0.04	15	0.19 ± 0.04	15	0.80			
		Contralateral	0.20 ± 0.04	15	0.20 ± 0.04	15	0.87			
		Ipsilateral	0.20 ± 0.04	15	0.19 ± 0.04	15	0.14			
		Diagonal	0.20 ± 0.04	15	0.19 ± 0.04	15	0.19			
	After multiple	Lame	0.20 ± 0.04	15	0.19 ± 0.04	15	0.67	0.19 ± 0.04	10	0.66
		Contralateral	0.20 ± 0.04	15	0.19 ± 0.04	15	0.42	0.19 ± 0.03	10	0.36
		Ipsilateral	0.19 ± 0.04	15	0.19 ± 0.04	15	0.98	0.19 ± 0.04	10	0.20
		Diagonal	0.20 ± 0.04	15	0.19 ± 0.04	15	0.35	0.19 ± 0.04	10	0.06
Swing time (sec)	Before	Lame	0.25 ± 0.04	15	0.25 ± 0.04	15		0.25 ± 0.04	9	
		Contralateral	0.25 ± 0.04	15	0.25 ± 0.04	15		0.25 ± 0.04	9	
		Ipsilateral	0.26 ± 0.03	15	0.26 ± 0.03	15		0.25 ± 0.03	9	
		Diagonal	0.25 ± 0.03	15	0.26 ± 0.04	15		0.24 ± 0.04	9	
	After single	Lame	0.25 ± 0.03	15	0.26 ± 0.03	15	0.26			
		Contralateral	0.25 ± 0.04	15	0.25 ± 0.04	15	0.19			
		Ipsilateral	0.26 ± 0.03	15	0.26 ± 0.03	15	0.86			
		Diagonal	0.26 ± 0.03	15	0.26 ± 0.03	15	0.08			
	After multiple	Lame	0.25 ± 0.03	15	0.25 ± 0.04	15	0.58	0.26 ± 0.04	10	0.80
		Contralateral	0.25 ± 0.04	15	0.25 ± 0.05	15	0.07	0.25 ± 0.04	10	0.33
		Ipsilateral	0.26 ± 0.03	15	0.25 ± 0.04	15	0.25	0.25 ± 0.03	10	0.63
		Diagonal	0.25 ± 0.03	15	0.26 ± 0.04	14	0.19	0.24 ± 0.03	10	0.39
Stride time (sec)	Before	Lame	0.44 ± 0.06	15	0.45 ± 0.07	15		0.43 ± 0.06	9	
		Contralateral	0.45 ± 0.06	15	0.45 ± 0.06	15		0.44 ± 0.07	9	
		Ipsilateral	0.45 ± 0.06	15	0.45 ± 0.06	15		0.44 ± 0.08	9	
		Diagonal	0.45 ± 0.06	15	0.45 ± 0.06	15		0.44 ± 0.07	9	
	After single	Lame	0.45 ± 0.06	15	0.45 ± 0.05	15	0.34			
		Contralateral	0.45 ± 0.06	15	0.45 ± 0.05	15	0.06			
		Ipsilateral	0.46 ± 0.06	15	0.45 ± 0.06	15	0.47			
		Diagonal	0.45 ± 0.06	15	0.45 ± 0.05	15	0.97			
	After multiple	Lame	0.45 ± 0.06	15	0.44 ± 0.06	15	0.28	0.44 ± 0.07	10	0.78
		Contralateral	0.44 ± 0.06	15	0.45 ± 0.07	15	0.25	0.44 ± 0.06	10	0.59
		Ipsilateral	0.45 ± 0.06	15	0.44 ± 0.06	15	0.29	0.44 ± 0.06	10	0.46
		Diagonal	0.45 ± 0.06	20	0.45 ± 0.07	15	0.24	0.44 ± 0.07	10	0.42
Stride length (cm)	Before	Lame	88.96 ± 14.37	15	89.17 ± 13.80	15		87.36 ± 14.20	9	
		Contralateral	88.90 ± 14.35	15	89.07 ± 13.91	15		86.05 ± 14.90	9	
		Ipsilateral	89.23 ± 14.09	15	88.94 ± 13.52	15		87.71 ± 13.86	9	
		Diagonal	89.57 ± 14.46	15	89.38 ± 13.95	15		87.44 ± 14.54	9	
	After single	Lame	88.22 ± 14.09	15	90.47 ± 14.77	15	0.52			
		Contralateral	87.84 ± 14.06	15	91.04 ± 15.19	15	0.13			
		Ipsilateral	88.70 ± 14.31	15	90.38 ± 14.53	15	0.90			
		Diagonal	88.41 ± 14.05	15	90.76 ± 14.75	15	0.49			
	After multiple	Lame	88.86 ± 13.45	15	90.14 ± 15.18	15	0.50	87.90 ± 14.36	10	0.96
		Contralateral	88.40 ± 13.41	15	92.60 ± 16.00	15	0.06	87.39 ± 14.24	10	0.66
		Ipsilateral	89.03 ± 13.56	15	90.68 ± 15.52	15	0.67	87.98 ± 14.17	10	0.96
		Diagonal	88.53 ± 13.32	15	93.04 ± 16.37	15	0.07	87.97 ± 14.26	10	0.81
Peak vertical force (%BW)	Before	Lame	61.15 ± 16.48	15	62.08 ± 16.56	15		59.33 ± 21.59	9	
		Contralateral	71.47 ± 20.97	15	72.57 ± 20.68	15		68.03 ± 23.22	9	
		Ipsilateral	67.33 ± 22.68	15	69.05 ± 22.84	15		70.71 ± 18.81	9	
		Diagonal	68.15 ± 22.52	15	70.85 ± 23.43	15		69.10 ± 18.99	9	
	After single	Lame	60.47 ± 17.37	15	58.94 ± 12.03	15	0.57			
		Contralateral	68.87 ± 20.03	15	68.98 ± 15.19	15	0.79			
		Ipsilateral	67.60 ± 23.60	15	69.48 ± 28.22	15	0.81			
		Diagonal	67.55 ± 23.25	15	69.40 ± 28.90	15	0.82			
	After multiple	Lame	62.16 ± 17.73	15	61.31 ± 17.61	15	0.70	55.40 ± 16.09	10	0.07
		Contralateral	73.00 ± 20.99	15	69.49 ± 20.55	15	0.40	61.19 ± 16.90	10	0.08
		Ipsilateral	72.03 ± 31.30	15	66.89 ± 22.89	15	0.26	70.55 ± 26.86	10	0.33
		Diagonal	73.53 ± 30.14	15	67.84 ± 25.56	15	0.15	69.85 ± 27.11	10	0.31
Vertical impulse (%BW*sec)	Before	Lame	7.16 ± 2.70	15	7.60 ± 3.19	15		6.38 ± 3.07	9	
		Contralateral	8.49 ± 3.44	15	8.90 ± 3.86	15		7.53 ± 2.80	9	
		Ipsilateral	7.59 ± 3.16	15	7.89 ± 3.26	15		7.80 ± 3.30	9	
		Diagonal	7.76 ± 3.08	15	8.18 ± 3.14	15		7.92 ± 3.46	9	
	After single	Lame	7.14 ± 2.71	15	7.00 ± 2.63	15	0.72			
		Contralateral	8.26 ± 3.04	15	8.24 ± 3.24	15	0.88			
		Ipsilateral	7.66 ± 3.20	15	7.67 ± 3.43	15	0.97			
		Diagonal	7.79 ± 3.24	15	7.76 ± 3.38	15	0.99			
	After multiple	Lame	7.36 ± 2.87	15	7.09 ± 2.74	15	0.75	6.18 ± 2.74	10	0.28
		Contralateral	8.89 ± 3.34	15	8.10 ± 3.27	15	0.28	6.85 ± 2.52	10	0.37
		Ipsilateral	8.31 ± 4.87	15	7.36 ± 3.03	15	0.33	7.98 ± 3.96	10	0.02 **
		Diagonal	8.54 ± 4.76	15	7.54 ± 3.55	15	0.26	8.05 ± 3.96	10	0.18

**Table 3:** Estimated mean values, number of dogs, and *p*-value for symmetry indices of peak vertical force (%BW). SD = standard deviation. N = number of dogs. %BW = percentage of body weight. SI = symmetry index. SI_limb_ = lame limb/sound contralateral limb. SI_sagittal_ = front- and hindlimb lame side/front- and hindlimb sound side. SI_transversefront_ = lame front limbs/sound hindlimbs. SI_transversehind_ = lame hindlimbs/sound front limbs. Single = measurement after a single treatment. Multiple = measurement after multiple treatments.

Parameter	Time	TENS (Mean ± SD)	N	Placebo (Mean ± SD)	N	*p*-Value	NSAID (Mean ± SD)	N	*p*-Value
SI limb Peak vertical force (%BW)	Before	0.87 ± 0.11	15	0.87 ± 0.11	15		0.87 ± 0.09	9	--
	After single	0.89 ± 0.11	15	0.86 ± 0.11	15	0.38			--
	After multiple	0.86 ± 0.12	15	0.90 ± 0.11	15	0.21	0.91 ± 0.07	10	0.07
SI sagittal Peak vertical force (%BW)	Before	0.92 ± 0.09	15	0.92 ± 0.09	15		0.95 ± 0.06	9	--
	After single	0.93 ± 0.09	15	0.93 ± 0.09	15	0.96			--
	After multiple	0.91 ± 0.10	15	0.94 ± 0.09	15	0.22	0.96 ± 0.06	10	0.52
SI transversefront Peak vertical force (%BW)	Before	1.64 ± 0.21	8	1.64 ± 0.24	8		1.67 ± 0.21	4	--
	After single	1.61 ± 0.23	8	1.63 ± 0.24	8	0.43			--
	After multiple	1.61 ± 0.19	8	1.61 ± 0.27	8	0.98	1.69 ± 0.31	4	0.05
SI transversehind Peak vertical force (%BW)	Before	0.57 ± 0.06	7	0.56 ± 0.07	7		0.55 ± 0.06	5	--
	After single	0.58 ± 0.06	7	0.56 ± 0.07	7	0.52			--
	After multiple	0.56 ± 0.06	7	0.57 ± 0.08	7	0.56	0.57 ± 0.06	6	0.98

## Data Availability

The datasets presented in this article are not readily available because the data are part of an ongoing study. Requests to access the datasets should be directed to anja.pedersen@slu.se.
